# A Recurrent Solitary Fibrous Tumor With an Exceptional Response to Low-Dose Radiotherapy: A Case Report and Literature Review

**DOI:** 10.7759/cureus.21199

**Published:** 2022-01-13

**Authors:** Toshiki Ishida, Toshiki Ohno, Takashi Saito, Yuichi Hiroshima, Shikama Akito, Iijima Tatsuo, Akihiko Yoshida, Masashi Mizumoto, Hideyuki Sakurai, Yoshio Tamaki

**Affiliations:** 1 Department of Radiation Oncology, Ibaraki Prefectural Central Hospital, Kasama, JPN; 2 Department of Endocrinology and Metabolism, Ibaraki Prefectural Central Hospital, Kasama, JPN; 3 Department of Pathology, Ibaraki Prefectural Central Hospital, Kasama, JPN; 4 Department of Diagnostic Pathology, National Cancer Center Hospital, Tokyo, JPN; 5 Department of Radiation Oncology, University of Tsukuba Hospital, Tsukuba, JPN

**Keywords:** huge, palliative, radiotherapy, sft, solitary fibrous tumor

## Abstract

Solitary fibrous tumor (SFT) is a soft tissue tumor derived from mesenchymal cells. We report a case of a giant SFT with insulin-like growth factor 2 (IGF-2) production in the pelvis of an 85-year-old male. SFT was diagnosed in surgery for a complaint of left lower abdominal distension. Subsequent tumor recurrence and progression caused rectal passage obstruction and hypoglycemia. Low-dose radiotherapy of 15 Gy in five fractions was started five years and four months after surgery, initially for a huge tumor around the rectum to improve rectal passage obstruction. The tumor volume shrank from 1054 cc before irradiation to 449 cc at one month and 396 cc at 10 months after irradiation. He had reached 90 years old at that time. Two months after the initial irradiation, similar radiotherapy of 15 Gy in five fractions was performed for a huge tumor in the right abdominal cavity. This tumor decreased from 1874 cc before irradiation to 615 cc at two months and 556 cc at seven months after irradiation. Dexamethasone (2.5 mg) was used for paraneoplastic syndrome at the time of initial radiation but was then reduced and became unnecessary two months after the second irradiation. Acute and late adverse events were mild. The patient is alive 60 months after the first irradiation. This case suggests that low-dose radiotherapy is beneficial as palliative therapy for symptom relief in patients with SFT.

## Introduction

Solitary fibrous tumor (SFT) is a rare soft tissue tumor derived from mesenchymal cells [1] that occurs frequently not only in the thoracic cavity but also in the retroperitoneum, head, and neck [2]. SFT is genetically defined by nerve growth factor-induced (NGFI)-A binding protein 2 (NAB2)-signal transduction and activator of transcription 6 (STAT6) fusion, which is correlated with the immunohistochemical nuclear overexpression of signal transduction and activator of transcription 6 (STAT6) [3]. Surgical resection is the main treatment option, and the five-year survival rates after complete resection are 96% and 68% for benign and malignant SFTs, respectively [4]. The recurrence rates for these SFTs are 2% and 16%, respectively, and malignant transformation often occurs at the time of recurrence [4]. Hypoglycemia due to insulin-like growth factor 2 (IGF-2) production from SFTs occurs in about 4% of cases as paraneoplastic syndrome [5]. Radiotherapy is mainly used postoperatively, but its role has not been clearly defined [2]. Here, we report a case of a giant SFT in the pelvis of a very elderly patient who was successfully treated with radiotherapy and had improved hypoglycemic symptoms.

## Case presentation

The patient was an 85-year-old male with a complaint of left lower abdominal distension. An examination revealed a mass lesion in the abdomen, which was diagnosed as SFT by surgery. At two years and eight months after surgery, there was a recurrence in the left paracolonic groove. The patient was followed up without treatment, but additional recurrence appeared on the right side of the bladder. Hypoglycemic symptoms also recurred at this time. The cause of hypoglycemia was thought to be paraneoplastic syndrome due to SFT, and dexamethasone was started.

**Figure 1:**
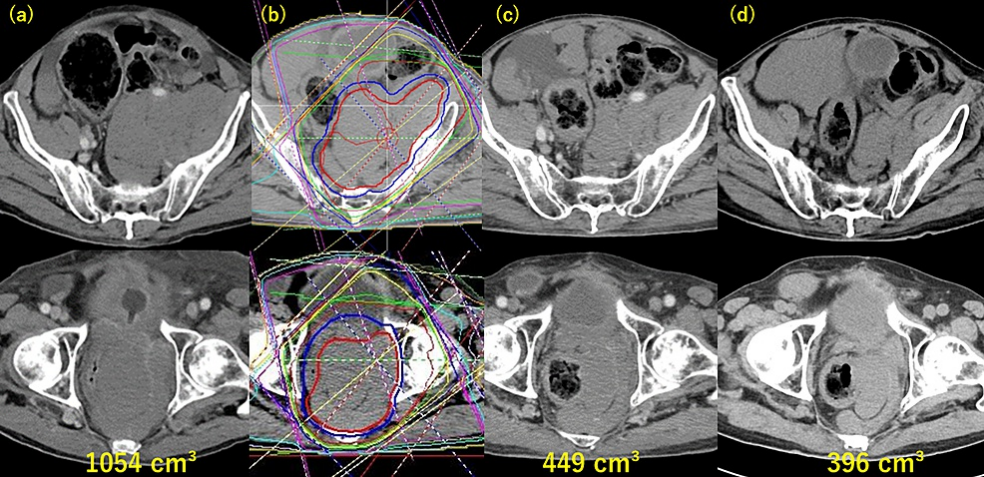
Tumor shrinkage after the initial radiotherapy. (a) Before radiotherapy (tumor volume: 1054 cc). (b) After palliative radiotherapy of 15 Gy in five fractions. (c) One month after radiotherapy (tumor volume: 449 cc). (d) Ten months after radiotherapy (tumor volume: 396 cc).

Due to tumor progression, rectal passage obstruction and uncontrollable hypoglycemia appeared, and radiotherapy was started at five years and four months after surgery. He had reached 90 years old at that time. Computed tomography (CT) showed a huge mass in the pelvis and right abdominal cavity, with rectal stenosis and obstruction of passage due to the tumor. It was difficult to use radiotherapy simultaneously for all lesions; thus, we decided to focus on the site of obstruction. Therefore, radiotherapy was initially performed on the huge tumor around the rectum to improve rectal passage obstruction (Figure 1). Given the age of the patient and the size of the lesion, low-dose radiotherapy (15 Gy in five fractions) was selected, with the possibility of additional irradiation if the response was poor. However, the tumor volume shrank from 1054 cc before irradiation to 449 cc at one month after irradiation and to 396 cc at 10 months (Figure 1).

**Figure 2:**
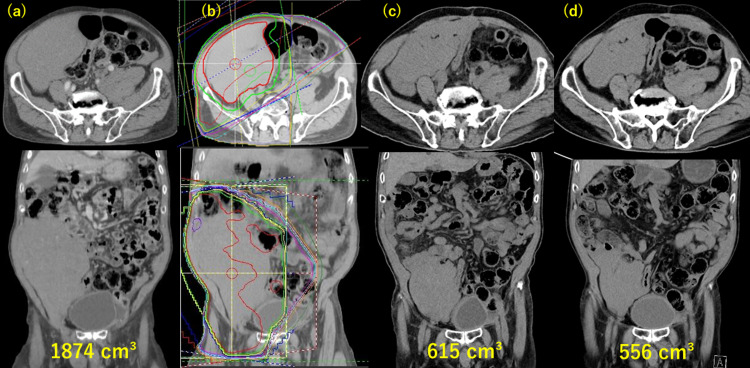
Tumor shrinkage after the second radiotherapy. (a) Tumor before the second radiotherapy (tumor volume: 1874 cc). (b) After palliative radiotherapy of 15 Gy in five fractions. (c) Two months after the second radiotherapy (tumor volume: 615 cc). (d) Seven months after the second radiotherapy (tumor volume: 556 cc).

Moreover, rectal passage obstruction was resolved. Two months after the initial irradiation, radiotherapy of 15 Gy in five fractions was performed for a huge tumor in the right abdominal cavity. The volume of this tumor decreased from 1874 cc before irradiation to 615 cc at two months after irradiation and to 556 cc at seven months (Figure 2).

**Figure 3:**
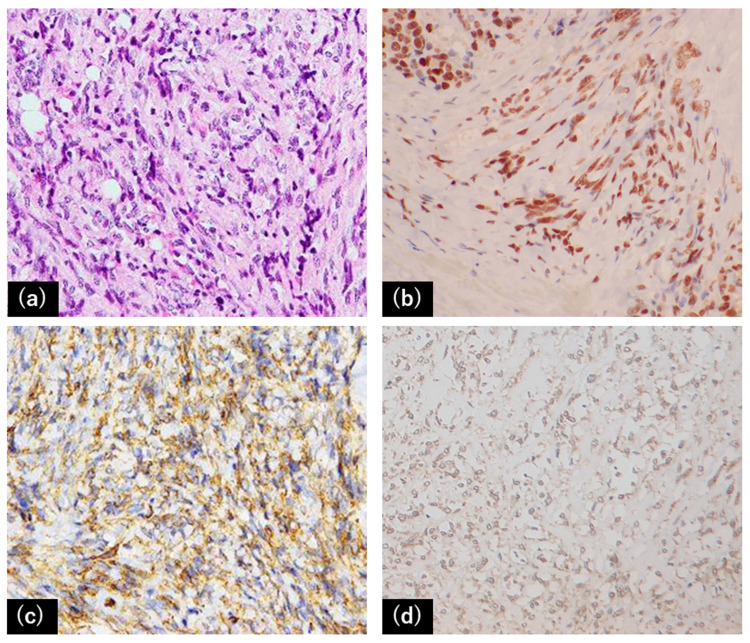
Pathological findings. (a) H&E staining showed proliferation of spindle tumor cells. (b,c,d) The spindle cells were immunoreactive for STAT6 (b), CD34 (c), and IGF-2 (d).

At the start of the first radiotherapy, 2.5 mg of dexamethasone was used for paraneoplastic syndrome. It was possible to reduce this dose gradually after the first irradiation, and dexamethasone became unnecessary two months after the second irradiation. Acute and late adverse events were mild (only grade 1 radiation enteritis occurred), and none of grade 2 or higher occurred during and after the courses of radiotherapy. The patient is still alive 60 months after the first irradiation. In the pathological findings of the first surgery (Figure 3), hematoxylin and eosin (H&E) staining showed proliferation of spindle tumor cells that were immunoreactive for STAT6, CD34, and IGF-2.

## Discussion

Hypoglycemia due to IGF-2 production from SFT is referred to as Doege-Potter syndrome [6]. Surgical resection is the main treatment for this syndrome, but about 70% of such cases have large tumors of >10 cm and are often difficult to resect [7]. Chemotherapy, embolization, and administration of steroid hormones are used for unresectable cases [8]; however, adverse events and side effects of the long-term use of steroids can become a problem.

There are only a few reports of radiotherapy for SFT. These include a 74-year-old female with a tumor of 11 cm maximum diameter who was treated with radiotherapy of 50 Gy in 25 fractions and survived for 12 months with tumor shrinkage of 36% [9], a 43-year-old male with a tumor of 22 cm maximum diameter who was treated with radiotherapy of 60 Gy in 30 fractions and survived for 10 months with tumor shrinkage of 44% [10], and a 66-year-old female with recurrence of SFT in the right lung who was treated with radiotherapy of 60 Gy in 30 fractions and had tumor shrinkage of 60% [11].

Our case involved a huge tumor in a 90-year-old super-elderly patient, and it was difficult to administer radiotherapy exceeding 50 Gy, as used in the above reports. As far as we are aware, there is no report of such good tumor shrinkage and improvement of hypoglycemic symptoms at a low dose of about 15 Gy in five fractions. Low-dose irradiation has advantages of a low risk of adverse events and a short irradiation period of only one week, compared to the usual period of five to six weeks for 50-60 Gy irradiation [12]. In addition, reirradiation can be considered when relapse or recurrence occurs near the irradiation field. However, this is the first report showing the efficacy of low-dose radiotherapy for SFT, and further verification of this effect is necessary.

## Conclusions

In conclusion, we experienced a case in which low-dose radiotherapy resulted in a marked reduction of the tumor and improvement of hypoglycemia in a case of SFT with IGF-2 production. This case suggests that low-dose radiotherapy should be considered as palliative therapy for symptom relief in patients with SFT.
